# Probability of success in the search for a related bone marrow donor in Cologne, Germany using HLA‐A, ‐B and ‐DRB1 haplotype frequencies

**DOI:** 10.1111/tan.13356

**Published:** 2018-09-21

**Authors:** Daniel M. Hellmann, Stela Radojska, Rolf Fimmers, Birgit S. Gathof

**Affiliations:** ^1^ Transfusion Medicine University Hospital of Cologne Cologne Germany; ^2^ Institute of Medical Biometry, Informatics and Epidemiology (IMBIE) University of Bonn Bonn Germany

**Keywords:** bone marrow donor search, haplotype frequency estimation, hematopoietic stem‐cell transplant, human leukocyte antigen, matched related donor, nuclear family

## Abstract

Between 2004 and 2013, 603 patients and their relatives (*n* = 1297) were typed as part of the search for a suitable HLA‐matched donor in their nuclear and extended families at the central service provider for transfusion medicine at the University Hospital of Cologne. The high success rate in finding donors over the years at our center (38.1%) led us to examine our database retrospectively in order to evaluate the donor search and haplotype frequencies (HFs) in the sample. Our goal was to identify the factors contributing to this high success rate and also to compare the HFs we observed with other reported haplotype frequency estimations (HFE) for the Cologne area. Probability estimations for a successful donor search were constructed based on the HFEs for the sample.

## 1 INTRODUCTION

Within the scope of allogeneic hematopoietic stem cell transplantation (HSCT), the search for suitable donors within the families of patients has many advantages over an unrelated donor search. Usually, family donors share complete haplotypes on a genetic level, which benefits the overall outcome of the bone marrow transplantation.^1^, ^2^, ^3^, ^4^ We observed that in most instances, family donors are highly motivated to help their relatives, and the timespan between the occurrence of the disease and the HLA typing is in general much lower than in the search for an unrelated donor. Average costs are also lower in many cases.^5^ When studying a collective of patients and their relatives who were typed for allogenic HSCT by transfusion medicine at the University Hospital of Cologne (North Rhine‐Westphalia, Germany), we observed few discrepancies regarding dubious paternity as well as high success rates over the years in finding HLA‐matched donors in the nuclear family. In light of the high social and cultural diversity in Cologne and the fact that 31% of residents have immigrant backgrounds,^6^ we sought to determine how this diversity would impact search results. An above‐average success rate has been reported in the Middle East, including developing Arabic countries, with success rates reaching 80%.^5^, ^7^, ^8^ The gap between reported success rates in the Middle East and the success rate experienced by our center motivated us to examine our database retrospectively. Our goal was to precisely estimate actual success rates in the search for donors within nuclear and extended families as well as to estimate the haplotype frequencies (HFs) for the sample in Cologne. This effort seemed particularly worthwhile in light of the fact that reliable data about the distribution of HFs in the population are important for donor databases in order to ensure that the recruiting of unrelated stem cell donors can be sensibly planned.^9^ Our overarching aim was to identify the causes of these high success rates in the family donor search and also to compare the HFs we observed with other reported haplotype frequency estimations (HFE) within the Central European population.

## 2 SUBJECTS AND METHODS

### 2.1 Properties of the sample

The sample we studied consisted of 1900 individuals, including 603 patients and their relatives (*n* = 1297) from consecutive family donors requiring allogenic HSCT. The sample was generated from January 2004 to April 2013 by the Transfusion Medicine Unit of the University Hospital of Cologne and analyzed retrospectively. Among the patients, 57.9% were male (*n* = 349) and 42.1% (*n* = 254) were female. The average age of the patients was 43.5 years (range: 0‐73 years). There were 59 (9.8%) pediatric cases (0‐18 years). Table 1 shows recruited donors and their degree of kinship and HLA compatibility to the patient. Within the scope of the analysis, compatibility was categorized as HLA‐identical, HLA‐haploidentical, 9/10‐match, 8/10‐match or no compatibility. In 18 of the family donor searches (3%), there was one or more haplotypes between the parents and their children that could not be explained by normal inheritance or a recombination of the alleles. These relatives were thus not representative of a family collective and were excluded from further analysis. For other families with surnames not of German ethnicity, we attempted to determine the ethnic origin of the patients by assessing their surnames. Determinations could not be made in all cases. Another approach was to determine the ethnicity by using ancestry‐informative markers.^10^ This was not possible in all cases due to the fact that we analyzed empirical data with ambiguous typing resolution. In light of this and the small sample size compared with other publications,^11^, ^12^, ^13^, ^14^, ^15^, ^16^, ^17^, ^18^ these families were kept in the analysis. It can be assumed, however, that depending on the location, the population being studied represents a cross‐section of the Central European population or is Caucasian.

**Table 1:** Recruited donors and their degree of kinship and HLA compatibility to the patient^a^

	HLA‐identical	HLA‐haploidentical	None of the same haplotypes	9/10 match	8/10 match	*n*
Brother	131	251	110	3	4	499
Sister	127	288	135	7	6	563
Father	2	58	—	1	—	61
Mother	1	60	—	1	1	63
Son	1	19	—	—	—	20
Daughter	—	10	—	—	—	10
Uncle	—	3	5	—	—	8
Aunt	—	7	8	—	—	15
Cousin	—	8	27	—	—	35
Cousin (female)	—	3	14	—	—	17
Nephew	—	1	2	—	—	3
Niece	—	1	1	—	—	2
Maternal half‐sister	—	1	—	—	—	1

a The table shows the familial relations between the patients in the sample and their HLA compatibility, both in the nuclear and extended family.

### 2.2 Statistical methods

All patient and donor data were initially entered into an Excel spreadsheet (Microsoft Corp., Redmond, Washington) and underwent statistical analysis with SPSS Statistics 22 (IBM Corp., Armonk, New York). The data entered into the spreadsheet included the HLA phenotypes and HLA ‐A, ‐B, ‐C, ‐DRB1 and ‐DQB1 loci of the patients and their family members; HLA compatibility and degree of kinship to the patients; as well as the age, gender and name initials of the sampled individuals. To derive the corresponding HFs, an expectation‐maximization (EM) algorithm (FAMHAP) was used, which iteratively calculates the maximum likelihood (ML) of the phenotypic HLA data and thus reconstructs where possible the four haplotypes of a nuclear family based on the data with an assumed Hardy‐Weinberg equilibrium (HWE).^19^, ^20^ To calculate the HFs, first‐degree family donors were consistently included for further investigation. To estimate the haplotypes of the nuclear families, some additional data had to be excluded from the analysis. This included datasets in which the familial relationships had not been clearly described or for which phenotypic HLA data for the loci (A‐B‐DRB1) examined was missing. The HFE was therefore based on 404 individual nuclear families including 1616 analyzed haplotypes. The HFs that could be determined in this way were compared with the HFs from other donor databases.

### 2.3 Quantitative and qualitative aspects of the data

In order to check the compatibility in our institute, we provided an initially low resolution typing at the HLA‐A, ‐B and ‐DRB1 loci in accordance with European Federation for Immunogenetics (EFI) standards.^21^ The HLA‐A and ‐B screening was performed on peripheral blood lymphocytes using the complement‐dependent cytotoxicity (CDC) crossmatch technique (BAG Histo Tray AB 144, BAG Health Care GmbH, Lich, Germany). The HLA‐DRB1 typing and in some cases (old blood samples, unclear serological typing results) the HLA‐A and ‐B typing were performed using the sequence‐specific‐primers (SSP) method (Olerup SSP HLA‐A, ‐B, ‐DRB1 low resolution kit, Olerup SSP AB, Saltsjöbaden, Sweden). HLA low‐resolution typing was performed by polymerase chain reaction SSP (PCR‐SSP) using the ABDR‐ and DR‐DQ Typing Tray (Olerup SSP AB). The high‐resolution testing was done with sequence‐based typing (SBT) (Celera Co., Alameda, California) and with SSP trays (Olerup SSP AB). For the DNA extraction from peripheral EDTA blood samples, the QIAamp DNA Blood Mini Kit (Qiagen GmbH, Hilden, Germany) was used. The confirmatory testing was provided by low‐resolution typing for the five loci HLA‐A, ‐B, ‐C, ‐DRB1 and ‐DQB1 if the haplotypes was ascertained by descent. HLA‐A, ‐B, ‐C, ‐DRB1 and ‐DQB1 high resolution typing was routinely performed when the identity couldn't be established by segregation. Due to the ambiguity of the phenotypic HLA data in terms of a heterogeneous typing resolution and the varying completeness of the phenotypic dataset available for the sample studied (HLA‐A: 99.67%, ‐B: 99.67%, ‐C: 53.16%, ‐DRB1: 98.21% and ‐DQB1: 54.51%), we encountered several problems associated with the use of the EM algorithm. Several other authors have encountered similar problems.^11^, ^12^, ^13^, ^14^, ^15^ These could be dealt with by tracing the HLA data back to a uniform serological or low‐resolution HLA nomenclature based on Version 3.2.0 of the IPD‐IMGT/HLA allele list.^22^ Serologically defined split antigens were traced back to their corresponding broad antigens. Furthermore, all DRB1 alleles were translated to a low‐resolution molecular genetic notation (two‐digit) in order to analyze the HFs. In light of the high number of absent antigens and alleles at the ‐C and ‐DQB1 HLA gene loci, only the HLA‐A, ‐B and ‐DRB1 loci were considered for the HFE.

## 3 RESULTS

Of 1216 individuals (1062 siblings, 124 parents and 20 children) from nuclear families, 262 HLA‐identical persons (258 siblings, 3 parents and 1 child) could be identified (Table 1). In terms of the donor search in nuclear families, an HLA‐identical donor could be found for 230 of the 603 patients (38.1%); 584 patients had at least one sibling in their nuclear family. Of those patients with one sibling, 228 patients could find at least one HLA‐matched sibling donor (39%). On average, 1.8 siblings per patient were typed.

Sibling compatibility could be broken down as follows: 258 HLA‐identicals (24.3%), 539 HLA‐haploidenticals (50.8%), 245 siblings with no HLA compatibility (23.1%), 10 9/10‐matches (0.9%) and 10 8/10‐matches (0.9%). Among the parents (*n* = 124), there were three HLA‐identicals (2.4%), two 9/10‐matches (1.6%) as well as one 8/10‐match (0.8%). An HLA‐identical donor could not be identified in the extended family donor search (*n* = 81). However, among the relatives in the extended family donor search, 29.6% shared at least one haplotype with the patient (*n* = 24).

From the existing data from the nuclear families, 658 haplotypes could be derived. The 239 haplotypes with a frequency of more than 1 in 1000 are described lexicographically and have a cumulative frequency of 71.92% (Table 2). The 20 most common HFs in our sample are detailed in Table 3.

**Table 2:** Distribution and frequencies of the HLA‐A‐B‐DRB1 haplotypes (HFs in 1000)^a^

A‐B‐DRB1	HF	A‐B‐DRB1	HF	A‐B‐DRB1	HF	A‐B‐DRB1	HF	A‐B‐DRB1	HF
1‐7‐15	1.99	2‐35‐04	2.05	3‐7‐07	1.38	11‐56‐01	2.04	26‐38‐04	1.51
1‐8‐01	2.08	2‐35‐08	1.80	3‐7‐11	2.06	11‐62‐04	1.54	26‐38‐13	3.40
**1‐8‐03**	**61.83**	2‐35‐11	2.00	3‐7‐13	1.54	23‐44‐03	1.36	26‐38‐14	3.93
1‐8‐04	2.77	2‐35‐13	2.79	3‐7‐14	2.71	23‐44‐04	1.35	26‐44‐11	1.36
1‐8‐07	1.37	2‐35‐14	3.81	**3**‐**7‐15**	**16.94**	23‐44‐07	6.11	26‐64‐14	1.36
1‐8‐11	3.30	2‐37‐11	1.11	3‐8‐03	1.28	24‐7‐01	1.37	29‐7‐10	2.04
1‐8‐14	1.37	2‐38‐13	2.72	3‐13‐07	1.36	24‐7‐04	1.39	29‐14‐07	1.36
1‐8‐15	6.18	2‐39‐16	1.36	3‐18‐11	4.40	24‐7‐15	6.50	29‐27‐04	1.36
1‐13‐07	2.97	2‐40‐04	1.36	3‐27‐11	1.36	24‐8‐03	2.15	29‐38‐04	2.04
1‐27‐04	1.10	2‐40‐11	1.36	**3‐35‐01**	**22.87**	24‐13‐07	2.03	**29‐44‐07**	**15.05**
1‐35‐11	1.33	2‐44‐01	4.22	3‐35‐03	1.21	24‐15‐11	1.36	29‐44‐15	1.44
1‐35‐13	1.36	**2‐44‐04**	**16.79**	3‐35‐04	4.09	24‐18‐11	4.70	29‐45‐04	1.36
1‐35‐14	1.36	2‐44‐07	5.23	3‐35‐08	1.36	24‐27‐01	2.04	30‐13‐07	8.81
1‐37‐07	1.40	2‐44‐11	4.87	3‐35‐11	3.83	24‐27‐11	1.54	30‐13‐15	2.05
1‐37‐10	3.39	2‐44‐12	2.76	3‐35‐13	1.38	24‐35‐01	1.94	30‐18‐03	2.72
1‐37‐15	1.36	2‐44‐13	6.87	3‐35‐14	1.84	24‐35‐04	2.04	30‐53‐03	2.04
1‐40‐13	2.04	2‐44‐14	2.79	3‐35‐15	2.24	24‐35‐07	1.62	31‐7‐15	1.36
1‐44‐07	5.38	2‐44‐15	4.68	3‐37‐03	1.36	24‐35‐11	7.04	31‐39‐15	1.36
1‐44‐11	1.28	2‐49‐11	1.36	3‐39‐16	1.36	24‐35‐12	1.36	31‐51‐01	1.27
1‐51‐11	2.16	2‐50‐07	3.26	3‐40‐03	1.36	24‐35‐13	5.25	31‐51‐14	1.36
1‐52‐11	1.33	2‐50‐11	1.36	3‐40‐11	1.36	24‐35‐15	1.41	32‐7‐01	2.04
1‐52‐15	3.15	2‐50‐15	2.85	3‐41‐13	2.72	24‐39‐08	2.03	32‐8‐03	1.40
**1‐57‐07**	**12.60**	2‐51‐04	2.16	3‐44‐07	1.10	24‐44‐01	1.36	32‐27‐13	1.37
1‐57‐13	1.38	2‐51‐08	1.50	3‐44‐11	2.98	24‐44‐12	2.91	32‐35‐11	1.36
1‐58‐08	1.36	2‐51‐11	8.02	3‐44‐12	3.16	24‐44‐15	1.61	32‐44‐03	1.37
1‐61‐14	2.04	2‐51‐12	1.35	3‐50‐03	1.36	24‐49‐04	1.02	32‐44‐13	2.04
1‐62‐04	4.61	2‐51‐13	4.32	3‐51‐04	1.21	24‐49‐11	1.02	32‐44‐16	1.36
2‐7‐01	3.41	2‐51‐15	1.36	3‐51‐11	2.09	24‐51‐04	2.60	32‐60‐04	1.36
2‐7‐03	1.37	2‐56‐01	3.39	3‐51‐13	4.42	24‐51‐08	1.21	32‐61‐11	1.65
2‐7‐04	3.28	2‐56‐15	2.04	3‐56‐01	1.36	24‐51‐15	3.54	32‐61‐14	1.07
2‐7‐12	1.38	2‐57‐07	7.54	3‐60‐04	2.04	24‐52‐15	1.36	33‐58‐03	2.04
**2**‐**7‐15**	**22.67**	2‐57‐13	2.05	3‐60‐11	1.36	24‐57‐07	1.36	33‐65‐01	2.03
2‐7‐16	1.36	2‐57‐15	1.25	3‐62‐04	2.72	24‐61‐11	1.36	33‐65‐11	1.36
2‐8‐03	4.13	2‐58‐04	2.04	11‐7‐13	2.30	24‐62‐11	1.12	66‐41‐13	2.04
2‐8‐13	1.50	2‐60‐04	2.84	11‐7‐15	1.57	24‐62‐13	1.36	68‐8‐03	1.03
**2**‐**13‐07**	**10.57**	2‐60‐07	1.41	11‐8‐03	2.75	24‐62‐15	1.96	68‐14‐13	1.36
2‐13‐15	1.36	2‐60‐08	2.13	11‐18‐03	2.04	25‐18‐01	1.35	68‐15‐04	2.04
2‐18‐03	2.84	2‐60‐13	7.83	11‐35‐01	7.46	25‐18‐04	1.35	68‐18‐11	1.26
2‐18‐04	1.46	2‐61‐04	2.04	11‐35‐04	3.87	25‐18‐15	4.76	68‐27‐15	1.36
2‐18‐11	7.35	2‐62‐01	2.21	11‐35‐07	1.25	25‐35‐11	1.67	68‐35‐03	1.02
2‐18‐15	2.14	2‐62‐03	1.37	11‐35‐14	2.71	25‐44‐01	2.03	68‐35‐11	1.36
2‐27‐01	2.04	2‐62‐04	6.63	11‐44‐04	1.51	25‐44‐11	1.36	68‐35‐13	1.35
2‐27‐04	2.40	2‐62‐07	1.43	11‐44‐11	1.51	25‐58‐13	1.36	68‐44‐01	1.36
2‐27‐07	1.40	2‐62‐11	2.10	11‐44‐14	2.26	26‐7‐15	2.04	68‐44‐11	2.72
2‐27‐08	1.23	2‐62‐13	5.17	11‐51‐11	1.50	26‐8‐03	1.37	68‐51‐09	1.36
2‐27‐11	3.89	3‐7‐01	1.86	11‐51‐15	1.84	26‐13‐07	1.36	68‐51‐13	3.22
2‐27‐16	2.72	3‐7‐03	2.88	11‐52‐04	1.36	26‐35‐01	1.36	68‐65‐13	2.04
2‐35‐01	7.87	3‐7‐04	2.02	11‐52‐15	2.04	26‐35‐11	1.36		

Abbreviation: HF, haplotype frequency.

a This table illustrates the 239 most common HLA‐A, ‐B and ‐DRB1 haplotypes in the sample with a frequency ≥1 in 1000. The table is arranged lexicographically by haplotypes and those haplotypes with a frequency ≥10 in 1000 are also shown in bold. The present data refer to the evaluation of the cohorts in the Transfusions Medicine Unit of the University Hospital of Cologne.

**Table 3:** Ranks and frequencies of the 20 most common HLA‐A‐B‐DRB1 haplotypes^a^

Frequency rank	HLA‐A	HLA‐B	HLA‐DRB1	HF in 1000
1	1	8	03	61.83
2	3	35	01	22.87
3	2	7	15	22.67
4	3	7	15	16.94
5	2	44	04	16.79
6	29	44	07	15.05
7	1	57	07	12.60
8	2	13	07	10.57
9	30	13	07	8.81
10	2	51	11	8.02
11	2	35	01	7.87
12	2	60	13	7.83
13	2	57	07	7.54
14	11	35	01	7.46
15	2	18	11	7.35
16	24	35	11	7.04
17	2	44	13	6.87
18	2	62	04	6.63
19	24	7	15	6.50
20	1	8	15	6.18

Abbreviation: HF, haplotype frequency.

a This extract illustrates the 20 most common haplotypes of the sample investigated.

## 4 DISCUSSION

What differentiates this study from most other HF estimates published to date is the fact that we only examined nuclear families to determine the HFs. With a frequency of 6.18%, the haplotype A1‐B8‐DR03 is the most common in our cohort and corresponds to the results from Schmidt et al^13^ (5.83%) and Eberhard et al^12^ (5.97%) for the German population as well as descriptions of the distribution of HFs in the Caucasian population^14^ (6.25%) within the National Marrow Donor Program (NMDP) as the most common haplotype in these reports overall. It is also the most common haplotype in the publication from Gourraud et al^23^ of the French Bone Marrow Donor Registry. Other common haplotypes in our sample are A3‐B35‐DR01 (2.29%), A2‐B7‐DR15 (2.27%), A3‐B7‐DR15 (1.69%) and A2‐B44‐DR04 (1.68%), which are also described as common in HFE reports for the Central European population.^24^ The individual position shifts in the frequencies—for example, compared with the reference data from Eberhard et al^12^—can be assessed by their larger sample size in relation to our cohort.

The empirical hit rate in the search for HLA‐identical donors among family members in our sample can be explained basically by the increased occurrence of sibling typing (with a ratio of 1.8 per patient in the overall sample in Cologne) and can be deduced as follows: Because the relevant alleles in the HLA system are closely coupled on the short arm of chromosome 6,^15^ the relevant haplotypes are generally inherited as a genetic unit. Theoretically, the likelihood of finding an HLA‐identical sibling among family members is quantified as 25%.^7^ To describe this hit rate more precisely, the rate at which the parents of the patients feature homozygous haplotypes or both parents are homozygous with each other must also be calculated. This could hypothetically be caused by accumulation of certain haplotypes in the gene pool of a population and a person inheriting the same haplotype twice by chance. This can be determined more precisely in the sample by deriving the rate of homozygosity of *m* = 0.0079. A less common reason for phenotypes of two siblings matching completely is chance matching of the phenotypes, even though different haplotypes were inherited from both parents. This probability corresponds to the chance that two unrelated persons have the same HLA phenotype and is calculated based on the HFs estimated by us of *k* = 0.000118. A formula for the probability F of finding an HLA‐identical sibling can be derived as follows:

F=0.25+0.5·m+0.25·k

The probability F that two siblings are HLA‐identical with one another is therefore 0.25 from the inheritance plus 0.5 multiplied by the homozygosity rate that one parent has the same haplotype twice, that is, he or she is homozygous, plus 0.25 multiplied by the rate k that the phenotypes between the siblings match by chance. This produces a probability of 25.4% for one sibling. The probability F therefore increases with each additional sibling according to the binomial distribution. The number of siblings in the sample varied from 1 to 7 siblings per patient (1*n* = 307, 2*n* = 165, 3*n* = 59, 4*n* = 31, 5*n* = 11, 6*n* = 8, 7*n* = 3). If the arithmetic mean is calculated taking the binomial distribution into account, according to the distribution of the number *n* of siblings per patient, this produces a theoretical hit rate F of 38.5%. If we now analyze the rate of successful stem cell donor searches in patients with at least one sibling (*n* = 584) in the family structure, we find an empirical hit rate of 39%, which most closely approximates the theoretical assumptions, taking into account statistical variations and possible recombinations and mutations of the HLA alleles. This formula can also be applied to extended family constellations (Table 4). For this purpose, the probability that two related persons with a degree of relatedness V have 0, 1 or 2 haplotypes in common due to inheritance is defined as: P(I = 0|V), P(I = 1|V) and P(I = 2|V). It has previously been shown that for siblings the following applies: P(I = 2|G) = 0.25, P(I = 1|G) = 0.5 and P(I = 0|G) = 0.25. The chance of a complete match between two random relatives is therefore calculated as:

$$P\left(I=2|V\right)+m\cdot P\left(I=1|V\right)+k\cdot P\left(I=0|V\right)$$

**Table 4:** Variable for calculating probability F for an HLA matched donor^a^

Degree of kinship	P(I = 0|V)	P(I = 1|V)	P(I = 2|V)
Sibling	0.25	0.5	0.25
Parent‐child	0	1	0
Uncle‐niece	0.5	0.5	0
Cousins	0.75	0.25	0

a The table lists various familial collectives with a view to probability F for an HLA matched donor in nuclear and extended families.

This formula shows that the probability of finding an HLA‐identical donor in the extended family donor search is extremely low. For cousins, the theoretical hit rate is 0.2%, and for aunts and uncles it is 0.4%. Between parents and their children, the chance of finding an HLA‐identical donor is 0.8%, according to the formula and the HFs in Cologne. Whether the extended family donor search produces results in light of this must be critically weighed. Our results show in this context that an extended family donor search was not able to achieve an HLA‐identical hit. Schipper et al^25^ have also described a formula for calculating the likelihood of finding a suitable donor in the extended family. They developed a software program (EXTFAM), to calculate the probability based on HFs, which is similar to our approach. The formula presented here is different, however, in that we draw on the total number of all HFs in order to calculate probabilities for discovering an HLA‐matched donor. Furthermore, using this formula we can simultaneously calculate the probability of finding a suitable donor in the nuclear family. With this formula, we seek to provide an overview of the general probability of finding a donor within the nuclear and extended family. Accordingly, the approach introduced in our study is more general, offering a broader overview of the likelihood of an HLA match, provided the HFs in the sample are known.

In sum, the results of our examination of data on HLA‐matched donor searches in Cologne show that higher success rates in our cohorts are strongly correlated with an increasing number of siblings, in accordance with the binominal distribution of HLA frequencies and considering the calculatory approach used in this study. Jawdat et al also come to the conclusion that it is not the age or sex of a patient, nor the quantity of consanguineous marriages in the population, but rather the number of siblings who are possible donors that plays a significant role in higher success rates.^7^ Furthermore, we could show that our HFEs largely correspond to the results obtained in other publications for the Caucasian population. This provides insight into the distribution of HFs in Cologne while also helping to optimize the quality of searches for related and unrelated bone marrow donors in the region.
